# Another Method of Banding a Logan Crown

**Published:** 1895-07

**Authors:** 


ARTICLE X.

### ANOTHER METHOD OF BANDING A LOGAN CROWN.

Dr. Thomas P. Hinman, of Atlanta, Ga., at the clinics of the Mississippi Association, demonstrated an original method of banding a Logan crown, without heating the crown for soldering the band. The unequal expansion of the large amount of metal in the Logan pin is liable to crack the porcelain, the metallic oxide formed in the process of heating, also changing the color of the crown. By

Dr. Hinman's new method, the root having been prepared as though for a Richmond crown, an open-mouthed band is fitted to the root and trimmed as desired to fit under the gum margin. Having the little screw-press of the Hollingsworth contouring method, the crown is held in the flame and heated red-hot, and the portion fitting the root burned in the block of wood belonging to the screw press, and a hole burned or reamed in the wood to receive the pin of the Logan crown. The crown is then placed in the band, with the pin in the hole, and forced down in place under the screw of the little press. The press has been improved by Dr. Hinman, the block of wood being made circular, fitting inside of the ring forming the floor of the press, and a swivel put on the point of the screw so that the crown is not twisted round and out of position, relative to its adjustment to the abutment of the root as the screw is turned down.

This makes a very tight fit of band to crown, requiring no cement; the band is cut away on the labial surface, allowing no gold to show on the face, but with heavy burnishers burnished closely over the palatine curvature. This method is adapted to the six anterior teeth. For bicuspids, Dr. Hinman cuts a slight groove, with a corundum stone, all around the crown, pressing the gold of the band closely with the groove.

Dr. Hinman is also at work on bridge-work made entirely of Logan crowns, doing away with the gold backings which so mar the beauty of porcelain teeth, one of the strong points of the method being that repairs in the mouth can be made with great speed, ease and perfection, though he thinks repairs will rarely be necessary.—*Items of Interest.*

